# Coralline algae in a naturally acidified ecosystem persist by maintaining control of skeletal mineralogy and size

**DOI:** 10.1098/rspb.2016.1159

**Published:** 2016-10-12

**Authors:** N. A. Kamenos, G. Perna, M. C. Gambi, F. Micheli, K. J. Kroeker

**Affiliations:** 1 School of Geographical and Earth Sciences, University of Glasgow, Glasgow G12 8QQ, UK; 2 Department of Integrative Marine Ecology, Stazione Zoologica Anton Dohrn, Villa Dohrn-Benthic Ecology Center, Villa Dohrn, Punta San Pietro 80077 Ischia, Naples, Italy; 3 Department of Biology, Stanford University, Hopkins Marine Station, Pacific Grove, CA 93950, USA; 4 Ecology and Evolutionary Biology, University of California Santa Cruz, CA 95064, USA

**Keywords:** crustose coralline algae, ecosystem, growth, mineralogy, ocean acidification, vents

## Abstract

To understand the effects of ocean acidification (OA) on marine calcifiers, the trade-offs among different sublethal responses within individual species and the emergent effects of these trade-offs must be determined in an ecosystem setting. Crustose coralline algae (CCA) provide a model to test the ecological consequences of such sublethal effects as they are important in ecosystem functioning, service provision, carbon cycling and use dissolved inorganic carbon to calcify and photosynthesize. Settlement tiles were placed in ambient pH, low pH and extremely low pH conditions for 14 months at a natural CO_2_ vent. The size, magnesium (Mg) content and molecular-scale skeletal disorder of CCA patches were assessed at 3.5, 6.5 and 14 months from tile deployment. Despite reductions in their abundance in low pH, the largest CCA from ambient and low pH zones were of similar sizes and had similar Mg content and skeletal disorder. This suggests that the most resilient CCA in low pH did not trade-off skeletal structure to maintain growth. CCA that settled in the extremely low pH, however, were significantly smaller and exhibited altered skeletal mineralogy (high Mg calcite to gypsum (hydrated calcium sulfate)), although at present it is unclear if these mineralogical changes offered any fitness benefits in extreme low pH. This field assessment of biological effects of OA provides endpoint information needed to generate an ecosystem relevant understanding of calcifying system persistence.

## 1. Background

A primary challenge for global change biology is to better understand the role of the sublethal effects of environmental change on individuals and populations in real world ecosystems. Laboratory experiments provide critical insight into the direct effects of environmental change on individual species, but it is often unclear how such effects will be balanced with other responses, or manifest in more complex ecosystems or naturally variable environments. Thus, a better understanding of the trade-offs among sublethal effects of environmental change, as well as how the sublethal effects reported in laboratory experiments materialize in field environments can improve forecasts of the ecological effects of global change.

Ocean acidification (OA), caused by the absorption of atmospheric carbon dioxide (CO_2_) into seawater, is projected to have widespread impacts on marine species and ecosystems in the near future [1]. Corals, coralline algae, foraminifera, sea urchins, molluscs and other marine organisms whose skeletons or shells are composed of calcium carbonate (CaCO_3_) are projected to be some of the worst affected, as changes in seawater chemistry may increase the energetic costs of building and maintaining CaCO_3_ structures [2,3]. In general, OA reduces growth [4], calcification [5] and abundance [6] of numerous calcifying species in laboratory experiments [7,8].

Coralline algae are important in ecosystem functioning and service provision; they create habitats and nursery areas, host high biodiversity, stabilize coral reef systems and contribute to carbon cycling [9–11]. Moreover, coralline algae are considered among the most sensitive species to OA due to the high magnesium (Mg) content of their CaCO_3_ skeleton [12]. The various polymorphs of CaCO_3_ utilized by marine calcifiers have different solubilities in seawater, and coralline algae are composed of the most soluble form of biogenic CaCO_3_: high-Mg calcite (7.7–28.8 mol% MgCO_3_) [13] with occasional aragonitic conceptacle infilling [14]. The solubility of calcite increases with its Mg content and high-Mg calcite is 50% more soluble than calcite and 20% more soluble than aragonite [15].

The negative effects of increasing *p*CO_2_ on coralline algae include reduced growth rates [16,17], calcification [18], epithelial integrity [19] and distribution/abundance [6,20–22]. Emerging research indicates that net growth or calcification of coralline algae can sometimes be maintained, or even increased, in elevated *p*CO_2_ conditions stimulated by day-time hypercalcification (rapid deposition of CaCO_3_) [13,23]. Field studies in naturally high *p*CO_2_ environments have found that some coralline algal species can grow at similar rates in ambient and high *p*CO_2_ conditions, and reductions in the abundance of coralline algae in these more acidic conditions may be driven by altered species interactions [24,25].

Continued skeletal accretion under high *p*CO_2_ conditions can come at the expense of skeletal structure [13,16,26] or a change in the skeletal mineral deposited [27]. A trade-off in skeletal structure for maintenance of growth or calcification could have indirect effects on coralline algal structure and abundance in high *p*CO_2_ environments. For example, OA-induced hypercalcification leads to CaCO_3_ skeletal material with poor molecular-scale ordering [13] and less stable cell walls [16]. In aragonitic calcifying algae, high *p*CO_2_ induced a change in skeletal mineralogy to less soluble calcium sulfates [27]. Such alteration in skeletal accretion could increase susceptibility to structural damage from grazing [26] or physical impacts [19]; for example, reduced thallus thickness in CCA may alter their competitive hierarchy and increase their susceptibility to grazing under more acidified conditions [26]. Therefore, while there is evidence that coralline algae can continue to calcify under OA [13,19], trade-offs can generate new sublethal effects, including calcified skeletons that are not as structurally coherent as those synthesized under current ambient conditions.

To understand the ecological effects of OA on coralline algae, we need to examine whether energetic trade-offs result in sublethal effects on CCA, which may in turn impact their abundance in an ecosystem. Observational field studies using natural gradients in environmental conditions provide opportunities to study the emergent effects of environmental factors on species and ecosystems. Such gradients incorporate environmental variability and species interactions that may mediate the sublethal effects of stressors [28]. In particular, shallow water volcanic CO_2_ vents create gradients in carbonate chemistry that have been used to study the effects of OA on coralline algae in the context of other co-occurring species. Previous studies have concluded that crustose coralline algae (CCA) from CO_2_ vents can maintain growth rates in low pH conditions, but it is unknown whether the maintenance of growth resulted in a trade-off with skeletal structure or masked other sublethal effects that limited the abundance of CCA in low pH [29]. Here, we investigate whether maintenance of CCA growth results in a trade-off with skeletal structure in a real-world, high *p*CO_2_, ecosystem context.

## 2. Material and methods

### (a) Location and experimental design

Research was conducted at the shallow volcanic CO_2_ vents (0.5–3 m depth) occurring on the north and south side of the small islet, Castello Aragonese at Ischia island (Italy; 40 43. 840′ N, 13 57. 080′ E). Gas was emitted at 0.7 × 10^6^ l day^−1^ in the north vent (area approx. 2000 m^2^) and 1.4 × 10^6^ l day^−1^ in the south site (area approx. 3000 m^2^) with no evidence of seasonal, tidal or diurnal variation in gas flow rates [20]. The north and south sites are 150 m apart, but are separated by a bridged road connecting the Castello islet to Ischia. At both sites, each pH zone was 20 m in length and was separated from the next zone by at least 20–25 m [20,30]. For full details of the site location and layout, see [20]. Water carbonate chemistry and *in situ* monitoring of seawater pH delineated a pH gradient with three carbonate chemistry zones (ambient, low and extreme low pH), caused by spatial variability in CO_2_ venting intensity [20]. In summary, the mean carbonate chemistry in the ambient pH zones corresponds to current average conditions (mean pH_North,South_ = 8.0, 8.1; mean saturation state of calcite_North,South_ = 4.8, 5.1), whereas the low pH zones are comparable with values predicted for 2100 (mean pH_North,South_ = 7.8, 7.8; mean saturation state of calcite_North,South_ = 3.0, 3.5) [30]. The carbonate chemistry in the extreme low pH zones approximates the most extreme acidification scenarios for the year 2300 [31] (mean pH < 7.4, mean saturation state of calcite less than 1.3). While there is more variability in carbonate chemistry of the low and extreme low pH zones, sampling with *in situ* pH sensors and discrete water samples across May–June 2010 [32], and September–October 2010 [30] reveals these zones were maintained within the standard deviations reported during these sampling periods. Regardless, it is not possible to attribute biological responses to particular carbonate chemistry values (e.g. mean pH in the low pH zones).

**Figure 1.**
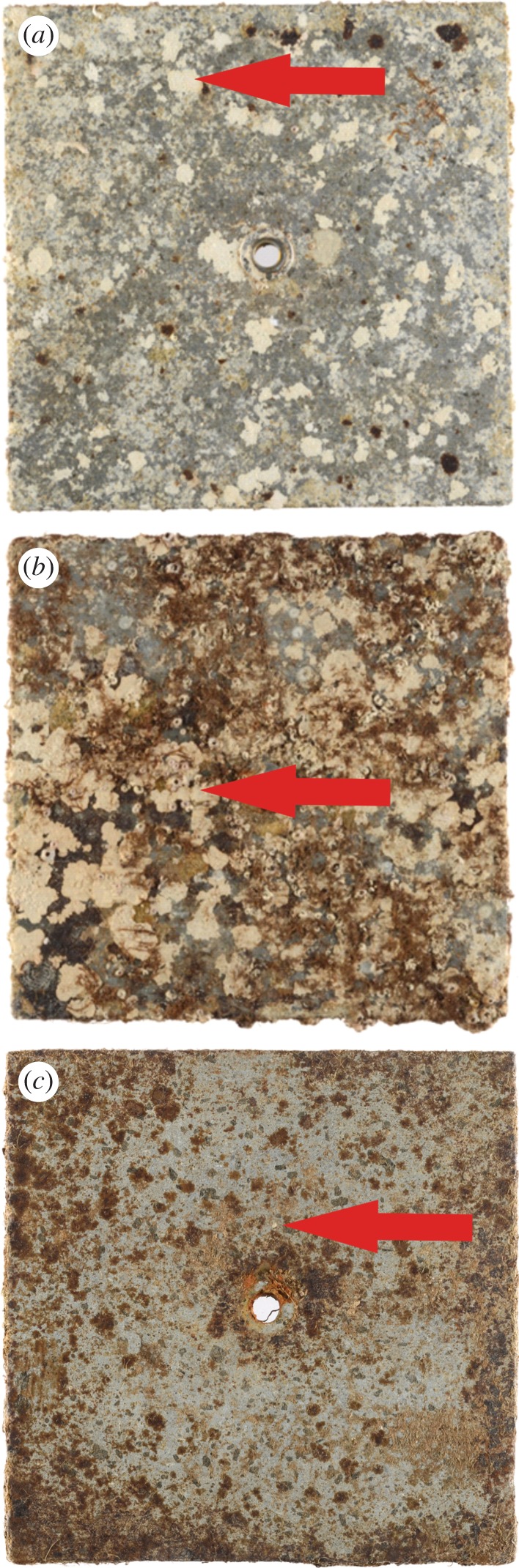
Benthic assemblages on exemplar tiles after 14 months exposure to CO_2_ vents at Castello Aragonese (Ischia island, Italy). (*a*) Ambient, (*b*) low and (*c*) extreme low pH zones from the south site. Arrows highlight some coralline algae individuals.

Nineteen recruitment stone tiles (15 × 15 × 1 cm) were attached to the substrate in each pH zone (ambient, low, extreme low pH) in both sites (north and south) at 1.5 m depth as part of a previous study [29] (figure 1). The tiles were made of volcanic stone from Vesuvium to provide a substrate and surface comparable to that of the natural surrounding environment. The tiles were bolted to the substrate in an approximately vertical orientation to allow the colonization of the local benthos. Independent tiles were collected from each site × pH zone over the course of the study at 3.5, 6.5 and 14 months. Once collected, tiles were preserved in 4% buffered formalin for 2 days and then stored in 70% ethanol. The CCA (live and dead; see discussion) from three genera (*Lithophyllum, Titanoderma* and *Phymatolithon*) growing on the tiles were identified under a dissecting scope using morphological features. All species showed the same responses (electronic supplementary material, table S1) so were pooled for subsequent analyses to enable statistically meaningful replication and interpretation.

### (b) Algal size

**Figure 2.**
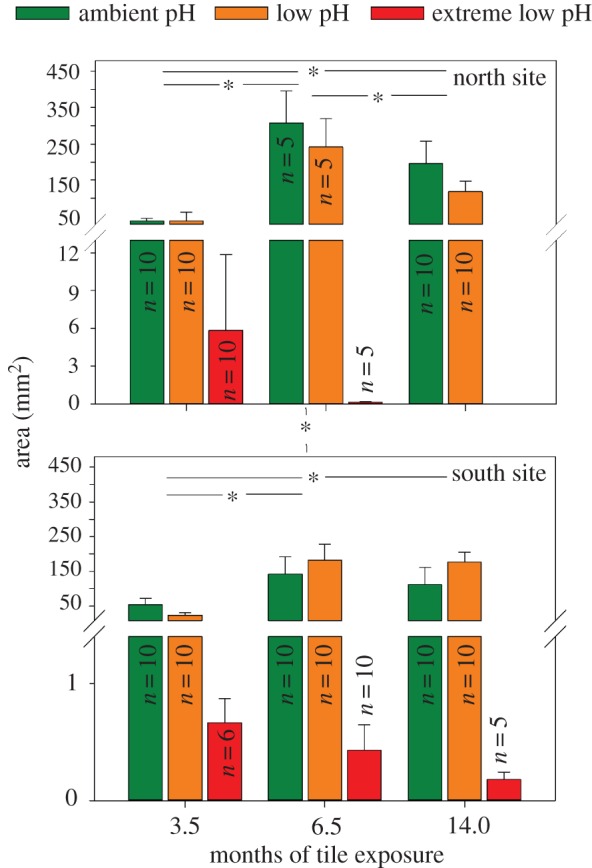
Coralline algal area. Area of the largest individual crustose coralline algae at the north and south sites growing on tiles collected after 3.5, 6.5 and 14 months from ambient, low and extreme low pH zones of CO_2_ venting zones at Castello Aragonese, Ischia island, Italy. Where available, the 10 largest CCA on each tile were used. Mean ± s.d. with significant differences indicated by asterisk.

Tiles were photographed (Nikon, D7000), and coralline algal size was determined using Image J (figure 1). The 50 largest individuals per tile were measured, when available. Of those, the 10 largest individuals measured on each tile were chosen for further comparison among pH zones and sites, as those will have been the oldest individuals; this was to avoid size underestimation driven by individuals that had settled on the tiles sometime after they were deployed (i.e. juvenile CCA settling on the tiles just before the 6.5 month measurement would have incorrectly reduced the size of the average individuals which had been growing for the full 6.5 month period). Where less than 10 individuals were present, all individuals were measured (see figure 2, for numbers available per treatment). In practice, very few individuals were present in the extreme low pH zones, resulting in only three to six individuals for each site × time point in extreme low pH.

### (c) Skeletal mineralogy and physical molecular structure

**Figure 3.**
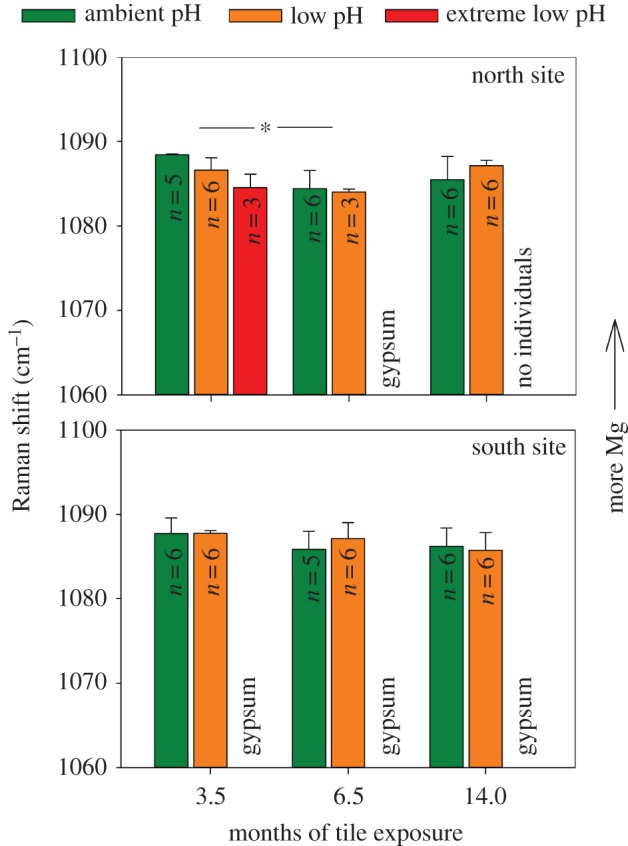
Frequency of the 1089 cm^−1^ Raman spectrum peak from crustose coralline algae. Higher frequency is equivalent to higher Mg concentration in coralline algae from the north and south CO_2_ venting sites at Castello Aragonese, Ischia island, Italy. Tiles containing algae were collected from ambient, low and extreme low pH zones at 3.5, 6.5 and 14 months after deployment. Mean ± s.d. with significant differences indicated by asterisk. Analyses not included where gypsum present.

Raman spectroscopy was used to investigate changes in the physical molecular structure of CCA that had settled and grown on the settlement tiles. Raman spectroscopy can be used to determine the skeletal mineralogy, quantify the relative Mg content, and the strength of the Mg–O bonds in calcite (below). Raman spectroscopy was carried out using Renishaw inVia Raman microscope equipped with a Leica DM 2500 M (Leica Microsystems GmbH, Wetzlar, Germany) microscope at the School of Geographical and Earth Sciences at the University of Glasgow (Glasgow, UK). CCA were analysed on the tiles and were excited using a 785 nm laser with a 1200 mm^−1^ grating via a 20× objective. Raman spectra were taken from individual CCA on six randomly selected tiles, ensuring all the genera were represented where possible. To remove organic material from the CCA surface, they were exposed to laser light prior to analysis for up to 60 s. However, fluorescence interference still caused some spectra to be rejected. Owing to the low abundance of CCA in the extreme low pH zone, only 20 Raman spectra were collected for extreme low pH (*n* = 3–6 per spectra treatment per time point per site, figure 3). The calcium mineral as well as the frequency and full width half maximum (FWHM, the peak width at half the peak height) of the approximately 1089 cm^−1^ peak were calculated using Wire v. 3.2 software.

### (d) Calcium mineralogy

Raman spectroscopy was used to identify the calcium mineral present by comparing the sample spectra with calcium minerals in the RRUFF library [33]. Calcite (ID: R040170), aragonite (ID: R040078) and gypsum (hydrated calcium sulfate (CaSO_4_ · 2H_2_O): ID: R040029) minerals are included in electronic supplementary material, figure S1, because of their presence in the samples.

### (e) Composition

The exact frequency of the 1089 cm^−1^ peak in spectra of biogenic calcite is primarily controlled by the Mg content (positive relationship) [13,34–36]. The frequency of the 1089 cm^−1^ peak increases with Mg concentration due to Mg ions being smaller than calcium ions, thereby causing a decrease in the interatomic distances and an increase in vibrational frequencies [35]. The exact frequency of the 1089 cm^−1^ peak (a proxy of relative Mg concentration) for each individual was extracted from the corresponding spectrum for analysis. Higher frequencies indicate more Mg within the high Mg skeleton. Where calcium sulfate was present as the skeletal mineral, this peak was replaced by a peak at approximately 1008 cm^−1^.

### (f) Carbonate ion positional disorder

Variation observed in bandwidth (FWHM) of the 1089 cm^−1^ Raman spectrum peak is due to the positional disorder of the carbonate ions in the calcite structure, which is characterized by increased rotation of the planar carbonate group out of the basal plane [37]. Specifically, positional disorder brings Mg and O ions closer together via Mg ions moving out of the plane parallel to the *a*-axis in the direction of the *c*-axis [35]. With Mg and O ions closer together, this generates a disordered lattice of Mg–O bonds [13,34]. Disordered lattices may be weaker than ordered lattices, probably making the skeleton more prone to breakage or other external damage [36]. The FWHM of the 1089 cm^−1^ peak for each individual was extracted from the corresponding Raman spectrum for analysis.

### (g) Statistical analyses

At 14 months, coralline algae were not observed growing on the tiles in the extreme low pH zone at the north site which prevented a balanced analytical design. Similarly, in the extreme low pH treatment, high Mg calcite was only present in CCA at 3.5 months. At all other time points where individuals were present the skeletal calcium carbonate mineralogy changed to gypsum, preventing direct comparison of the mineralogy. Thus, extreme low pH zones were excluded from statistical analysis for Mg composition, carbonate ion positional disorder and area. Analyses of variance (general linear model) with Tukey's pairwise comparisons were used to determine differences in Mg composition, carbonate ion disorder and areas between time and pH zone (both fixed) with site as a random variable with interactions being examined. Area was log_10_ transformed to meet general linear model assumptions. Time was not treated as a repeated measure as at each time point separate tiles were collected and removed, thus individuals were not re-sampled.

## 3. Results

### (a) Size

Despite reductions in abundance of CCA in low pH zones compared to the ambient pH zones [29], we did not detect differences in the size of the 10 largest individual patches between these two zones (*F*_1,100_ = 3.41, *p* = 0.068; figure 2). In both north and south sites, CCA were significantly larger at 6.5 and 14 months than at 3.5 months, however in the south site, CCA at 14 months were smaller than those at 6.5 months (*F*_2,100_ = 177.33, *p* < 0.001 with Tukey's HSDs *p* < 0.002 were significant). These differences generated significant interactions between month and site (*F*_2,100_ = 4.11, *p* = 0.019) as well as month and pH zone (*F*_2, 100_ = 5.53, *p* = 0.005) but not site and pH (*F*_1,100_ = 3.64, *p* = 0.069). At 6.5 months, CCA in the north site were significantly larger than those in the south site (Tukey's HSD *p* = 0.01). Few coralline algae were present in the extreme low pH zones (figure 2). Although it was unclear if they were alive or dead at the time of the collection, we include all individuals as a snapshot of fitness and mineralogy in extreme low pH.

### (b) Skeletal mineralogy

During all sampling time points at both ambient and low pH zones, the skeletal material of all CCA was composed of calcite (high Mg) or a mix of calcite (high Mg) and aragonite (electronic supplementary material, figure S1). However, in the extreme low pH zones, CCA were only composed of high Mg calcite in the north site at 3.5 months. CCA in the extreme low pH zones of the north site at 6.5 months and south site at 3.5, 6.5 and 14 months had skeletal material composed of a gypsum mineral. In the north extreme low pH sites, CCA were not present on the tiles at 14 months (electronic supplementary material, figure S1).

### (c) Mg Concentrations

Mg concentrations (peak frequency of the 1089 cm^−1^ Raman spectrum peak) of CCA calcium carbonate did not differ between ambient and low pH locations (*F*_1,57_ = 0.07, *p* = 0.795; figure 3) or between north and south sites (*F*_1,57_ = 2.51, *p* = 0.118). In the north site, Mg concentrations were significantly lower at 6.5 months than 3.5 months (*F*_2, 57_ = 7.83, *p* = 0.001 with Tukey's HSDs *p* = 0.008). There were no significant interactions between month and site (*F*_2,57_ =2.49, *p* = 0.092), month and pH (*F*_2,57_ = 1.03, *p* = 0.363) or site and pH (*F*_1,57_ = 0.08, *p* = 0.774).

### (d) Carbonate ion positional disorder

**Figure 4.**
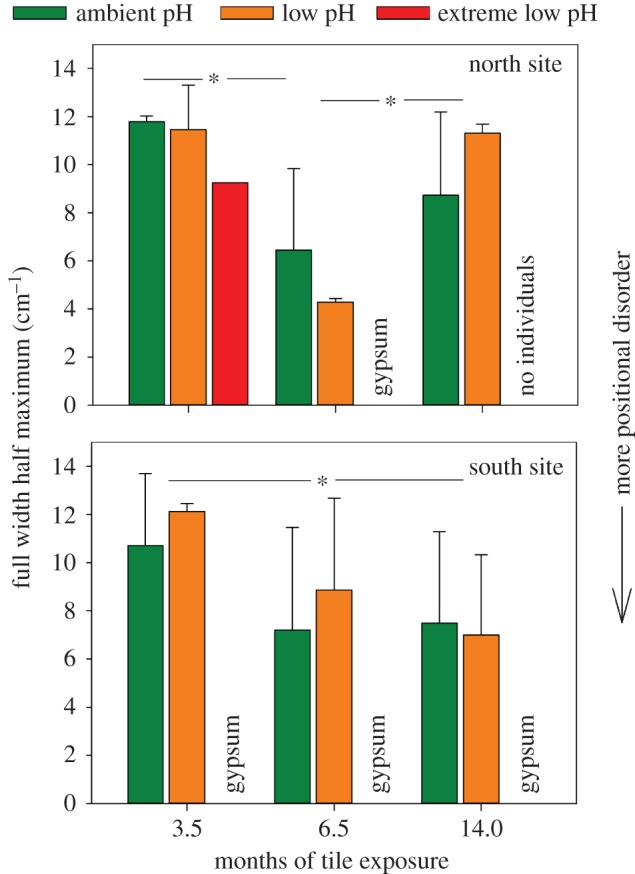
Full width half maximum (FWHM) of the 1089 cm^−1^ Raman spectrum peak from crustose coralline algae (see figure 2 for numbers analysed per treatment). Higher FWHM is equivalent to lower positional disorder in skeletal calcium carbonate deposited by coralline algae from the north and south CO_2_ venting sites at Castello Aragonese, Ischia island, Italy. Tiles containing algae were collected from ambient, low and extreme low pH zones at 3.5, 6.5 and 14 months after deployment. Mean ± s.d. with significant differences indicated by asterisk. Analyses not included where gypsum present.

Positional disorder (FWHM of the 1089 cm^−1^ Raman spectrum peak) of CCA calcium carbonate did not differ significantly between ambient and low pH locations (*F*_1,57_ = 0.52, *p* = 0.475, figure 4) or between north and south sites (*F*_1,57_ = 0.04, *p* = 0.847). In the north site, positional disorder was lower at 3.5 and 14 months than 6.5 months and in the south site positional disorder was lower at 3.5 months than 14 months (*F*_2,57_ = 15.26, *p* < 0.001, with Tukey's HSDs *p* < 0.005 were significant).

## 4. Discussion

Individual CCA patches growing in low pH/high *p*CO_2_ conditions similar to those projected for 2100 (i.e. the low pH zones of the Castello vents) can maintain growth and calcification without detectable trade-offs in skeletal structure. The size of the largest CCA patches did not differ between ambient and low pH conditions over 14 months, suggesting growth of some individuals can be maintained in low pH (mean 7.8). This unexpected result is in agreement with growth measurements taken during the early stages of succession using repeated measurements on discrete patches of CCA over 3.5 months [29], as well as with observations on algal recruitment over shorter timescales [38]. While results presented here suggest that some CCA patches could maintain their growth and skeletal structure in low pH, a previous study [29] found the total per cent cover of CCA was significantly less in low pH than ambient pH. Our *in situ* results indicate that the maintenance of growth of these individual CCA patches did not come at the expense of sublethal changes in skeletal structure, suggesting that these individuals may be already acclimated/adapted to these low pH conditions. The reduced CCA per cent cover observed in [29] could signal that CCA were limited in low pH by other factors such as altered interactions with competitors and herbivores [29], or that while some individuals continued to grow, others were more sensitive to pH changes, or increased dissolution in low pH, resulting in lower per cent cover. Importantly, if we consider the ability for some individuals to continue growing in lower pH, at longer timescales, they may provide the recruits for CCA populations capable of surviving in lower pH environments of the future. Such high variability in individual responses to OA has been reported in CCA [26] and other systems [39,40].

The calcium mineral composing the CCA skeleton in the ambient and low pH zones was calcite or calcite with some aragonite. Similarly, there was a general trend of no difference in the Mg content and skeletal molecular-scale structural disorder of the CCA grown in ambient and low pH zones, suggesting these temperate CCA can continue to accrete high Mg calcite at low pH levels projected for the turn of this century. This also occurs in the surface of tropical CCA, which maintain their Mg concentrations in reduced pH [41]. The presence of some aragonite in the skeleton is a natural process occurring where there has been skeletal exposure to seawater [42] or in the conceptacles [14] and is thus not likely associated with the site or age of growth.

This study, Diaz-Pulido *et al*. [41] and Nash *et al*. [43] suggest that CCA are not reducing their Mg concentrations to make their calcite less reactive. However, other investigations [15,16,44] including our own [13,36] have reported a reduction in the Mg content of coralline algae with OA. It is unclear if maintenance of CCA Mg content in individuals from low pH in this system is due to their ability to buffer (internally or externally) against changes in seawater carbonate chemistry or if the carbonate chemistry the CCA were exposed to in the low pH zones was not as physiologically or thermodynamically stressful as laboratory conditions. This could be because pH at the Ischia vents is variable through time due to the effect of winds, tides and day–night productivity cycles [30], and pulsed exposure to low pH appears not to alter Mg content (this study and [45]), as opposed to the prolonged stable exposures often used in laboratories.

Although high Mg calcite becomes less stable in low pH, it is unknown if incorporating and maintaining Mg in the calcite lattice is more energetically costly in low pH conditions. Moreover, little is known about the seawater chemistry in the diffusive boundary layer of CCA at this site. It is thus possible that CCA regularly experience variable pH/high *p*CO_2_ conditions in the diffusive boundary layer due to (i) respiration at night, and (ii) conversely, it is possible that turf algae in close association with CCA could locally elevate seawater pH in the diffusive boundary layer during photosynthesis to buffer the CCA from the acidic waters in the low pH zone [46,47]. If the latter does occur, turf algae that are more abundant in the low pH zone than the ambient pH zone [29] could potentially ameliorate the chemistry locally. Understanding the emergent effects of turf algae on CCA abundance, however, requires consideration of other effects, such as shading. More research is needed to better understand (i) how CCA incorporate Mg in ambient conditions and (ii) the mechanisms for the maintenance of high Mg calcite in CCA grown in low pH.

In contrast with the CCA in low pH, those found in extreme low pH were rare and very small. This suggests they are (i) selected against by critically low pH levels, (ii) grow more slowly or (iii) are affected by other post-settlement processes (including faster dissolution) and therefore play a reduced ecological role in these conditions. Their presence, however, allowed us to better understand how the mineralogy, Mg content and structural disorder of CCA can change in extreme pH conditions. In the extreme low pH zone, the skeletal mineral changed from calcite or calcite and aragonite in the ambient and low pH zones to gypsum by either 3.5 (South) or 6.5 (North) months. Gypsum contains no Mg and is more stable than calcite or aragonite in low pH [27]. As we did not observe individuals that contain both gypsum and calcite/aragonite, this may signal some individuals have the ability to switch directly from one mineral to the other when environmental conditions change. Similar changes in the calcium mineral also occur in the calcifying alga *Padina pavonica,* which changes from aragonite to gypsum in low pH [27]. Other organisms also deposit gypsum including desmids [48,49], medusa [50,51] and microorganisms [52]. So along with post-depositional alteration to gypsum, there is also a precedent for native biomineralization using gypsum in marine systems. It should be noted that post-mortem replacement of calcite by gypsum can occur, but this is at geological timescales and in fossil material [53] and thus was not a likely process in CCA from the extreme low pH zone, where changes occurred in less than 6.5 months. Thus, algal deposition of a gypsum skeleton may be attributed to phenotypic plasticity that allows species to exist in variable environments, the linkages between algal biology and biomineralization, and possible photosynthetic benefits algae may obtain from higher *p*CO_2_ [27,54]. Despite the increased stability of gypsum in low pH when compared with calcium carbonate, it is unclear whether this change increased the fitness of the individuals in extremely low pH because it is unknown whether the individuals were alive or dead at the time of collection. Regardless of the status of the CCA in extremely low pH, this result suggests a potential mineralogical response that may provide a fitness benefit to CCA in more moderate pH conditions.

The variability in the responses of extreme low pH CCA between sites and among time points restricts our ability to make robust conclusions about the impacts of extreme low pH, but it also provides an opportunity to develop hypotheses about the potential drivers of variability. We cannot determine the physiological condition of the CCA from the extreme low pH at the time of collection (e.g. whether these CCA were healthy but small, or whether these samples were already dead and dissolving). If the CCA were dead, they may have not survived in the extreme low pH treatment despite the mineralogical changes. However, there is evidence that live calcifying algae can change their calcium mineral [27]. Thus, if the CCA were alive, the major change in calcium mineral present in the extreme low pH suggests there could be a threshold pH, which occurs somewhere between the low (mean pH 7.8) and extreme low pH (mean pH 6.9), where calcium minerals with no or low Mg become beneficial to the organism. If this and similar responses in *Padina pavonica* [27] are coping mechanisms, it is possible that even at extremely low pH, via a bio-induced change in their skeletal calcite mineral, coralline algae may still be able to calcify albeit with substantially reduced growth and abundance.

In the extreme low pH zone in the south site, the gypsum is present throughout the time series. By contrast, the gypsum only appears in the extreme low pH zones in the north site at 6.5 months. If the CCA were alive, this inconsistency among sites could potentially be due to differences in the timing of CCA recruitment to the tiles at each site. For example, recruitment may have happened earlier in the south than the north. Differences in the age of the recruits would correspond to different lengths of CCA exposure to extreme low pH, which could explain the site differences. Future studies with higher temporal resolution of the Mg content of CCA recruits can help test this hypothesis.

It is important to note that the reactivity of calcium minerals to their environment is not solely controlled by the concentration of Mg in their skeletons. Rather, the reactivity of the calcium minerals post deposition is driven by a combination of (i) the saturation state of the surrounding fluid or water, (ii) the microstructural complexity of the biomineral, and (iii) the mineral stabilities [55]. It is thus possible that by changing their skeletal calcite mineral, CCA are also changing the microstructural stability of their biomineral, making them more or less susceptible to the saturation state of the surrounding water [55].

Environmental variability between the north and south could contribute to the differences seen in the responses of area, calcite mineral, Mg content and positional disorder to pH between the north and south sites at different time points (also indicated in the significant, but not consistent, statistical interaction terms). For example, the north and south sites are characterized by different exposures to light intensity, dominant winds, wave action and fetch. Generally, the south site is more sheltered and the pH gradient is more consistent [30]. Because the north site is often more exposed to wave action than the south, we hypothesize that the ambient and low zones could have been bathed in lower pH water from the extreme low pH zones due to wave action prior to the tile collection at particular times, which could have affected the skeletal deposition across all three pH zones.

We observed no differences in response between the different genera of CCA (electronic supplementary material, table S1) to individual site pH zone-time points. It may be that this apparent equivalency among taxa is due to the relatively low numbers of available individuals from each genus (total individuals across all genera = 3–10 per sampling unit) causing low statistical power. However, if this pattern holds across larger populations of individuals from particular genera, it is possible that the estimated millennial longevity of the venting systems at Castello Aragonese [56] has been long enough to allow different CCA species to adapt similarly to acidified conditions. This is in contrast with genus and species specific CCA responses [24,26] demonstrated in areas where *p*CO_2_ enrichment is a more recent process [57] and adaptation/acclimatization processes may still be ongoing. Similar responses among different species of CCA would suggest that competitive hierarchies and species interactions may also be resilient to changing ocean conditions in some scenarios.

## 5. Conclusion

CCA present in a naturally acidified ecosystem appear to be skeletally robust to the *p*CO_2_ enrichment projected for 2100, although their abundance is probably limited by their interactions with other species at these *p*CO_2_ levels [29]. In more extreme conditions, the size of CCA was greatly reduced and the mineralogy was altered, which may have been a coping mechanism by the CCA to reduced pH while alive. To fully understand the long-term persistence of coralline algae in a changing world, a better understanding of (i) how mineralogical responses might contribute to CCA resilience to OA and (ii) why some CCA change their Mg content or skeletal mineralogy with OA are needed.

## Supplementary Material

Kamenos et al supp mat 25_08_16.docx ( 355K )

## Data accessibility

Data are available online from Dryad: http://dx.doi.org/10.5061/dryad.6140t [58].
