# Supplementary material for: Coralline algae in a naturally acidified ecosystem persist by maintaining control of skeletal mineralogy and size
Source: Proc Biol Sci. 2016 Oct 12;283(1840):20161159. doi: 10.1098/rspb.2016.1159 (PMC5069505; doi:10.1098/rspb.2016.1159)
Supplement: Kamenos et al supp mat 25_08_16.docx ( 355K ) [file rspb20161159supp1.docx]

**Coralline algae in a naturally acidified ecosystem persist by maintaining control of skeletal mineralogy and size**

Kamenos, N.A., Perna, G., Gambi M.C., Micheli, F. and Kroeker K.J.


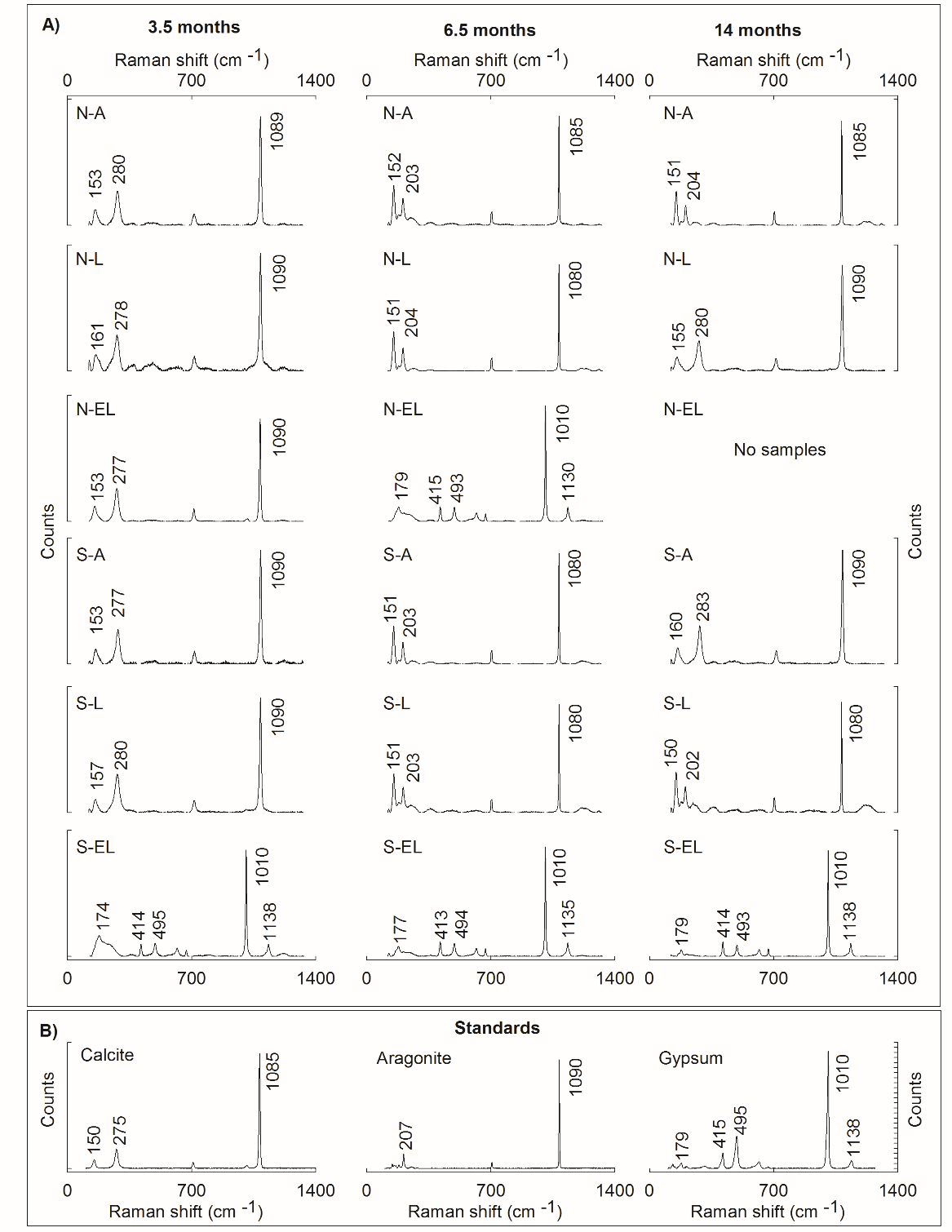


**Figure S1:** Raman spectra A) Representative Raman spectra from representative coralline algae growing on settlement tiles in North (ambient, low & extreme low pH (N-A, N-L & N-EL respectively)) and South sites (ambient, low & extreme low pH (S-A, S-L & S-EL respectively)) of Castello Aragonese at 3.5, 6.5 and 14 months post tile deployment. B) Raman spectra of Calcite (RRUFF ID: R040170), Aragonite (RRUFF ID: R040078) and Gypsum (calcium sulphate dihydrate) (RRUFF ID: R040029) standards from the RRUFF database [1]. Vertical numbers associated with spectra indicate peak positions.

**Table S1:** Genus-specific area, frequency and full width half maximum (FWHM) from North (ambient, low & extreme low pH (N-A, N-L & N-EL respectively)) and South sites (ambient, low & extreme low pH (S-A, S-L & S-EL respectively)) of Castello Aragonese at 3.5, 6.5 and 14 months post tile deployment. Mean ± SD for each genus at each site-pH zone-time point presented and compared with a Kruskal-Wallis (H, P, df) where individuals present. Missing cells indicate no individuals present, or for statistical results, only one genus present.

|  | Variable | *Lithophyllum sp.* | *Titanoderma sp.* | *Phymatolithon sp.* | H / P / df |
| --- | --- | --- | --- | --- | --- |
| N-A-3.5 | Area (mm^2^)  Frequency (cm^-1^)  FWHM (cm^-1^) | 42.5 ± 8.2  1087.9 ± 0.5  11.9 ± 0.6 | 33.1 ± 4.8  1088.5 ± 0.2  11.9 ± 0.1 | 38.5 ± 7.5  1088.4  11.9 | 2.90 / 0.407 / 2  3.60 / 0.165 / 2  0.00 / 1.000 / 2 |
| N-A-6.5 | Area (mm^2^)  Frequency (cm^-1^)  FWHM (cm^-1^) | 395.2 ± 63.7  1083.2 ± 1.4  11.3 ± 0.2 | 261.7 ± 66.5  1085.5 ± 8.1  8.2 ± 4.9 | 219.9  1084.2  4.2 | 3.60 / 0.165 / 2  1.14 / 0.565 / 2  1.14 / 0.565 / 2 |
| N-A-14 | Area (mm^2^)  Frequency (cm^-1^)  FWHM (cm^-1^) | 240.7 ± 71.1  1087.8 ± 0.2  11.3 ± 0.2 | 167.3 ± 37.5  1086.1 ± 2.8  7.6 ± 5.1 | 164.8 ± 43.1  1082.6 ± 1.3  7.3 ± 3.9 | 2.93 / 0.231 / 2  3.43 / 0.180 / 2  2.00 / 0.368 / 2 |
| N-L-3.5 | Area (mm^2^)  Frequency (cm^-1^)  FWHM (cm^-1^) | 38.3 ± 24.8  1087.5 ± 0.4  12.2 ± 0.1 | 27.7 ± 28.5  1087.1 ± 0.2  12.3 ± 0.2 | 42.6 ± 27.5  1085.3 ± 2.3  9.9 ± 3.1 | 0.64 / 0.727 / 2  4.57 / 0.102 / 2  3.43 / 0.180 / 2 |
| N-L-6.5 | Area (mm^2^)  Frequency (cm^-1^)  FWHM (cm^-1^) | 277.4 ± 123.3  1084.2  4.1 | 226.6 ± 63.5  1084.2  4.3 | 199.3  1083.6  4.4 | 0.40 / 0.819 / 2 |
| N-L-14 | Area (mm^2^)  Frequency (cm^-1^)  FWHM (cm^-1^) | 143.1 ± 22.9  1087.8 ± 0.2  11.7 ± 0.3 | 101.1 ± 11.1  1086.9 ± 0.6  11.1 ± 0.3 | 114.8 ± 37.5  1086.8 ± 0.1  11.2 ± 0.3 | 3.84 / 0.147 / 2  3.43 / 0.180 / 2  3.71 / 0.156 / 2 |
| N-EL-3.5 | Area (mm^2^)  Frequency (cm^-1^)  FWHM (cm^-1^) | 8.9 ± 6.4  1083.4  9.3 | 6.1 ± 6.0  1085.7  9.3 | 0.8 ± 0.2 | 2.95 / 0.229 / 2  1.00 / 0.317 / 1 |
| N-EL-6.5 | Area (mm^2^)  Frequency (cm^-1^)  FWHM (cm^-1^) | 0.2 ± 0.1 | 0.1 ± 0.1 | 0.1 | 3.60 / 0.165 / 2 |
| N-EL-14 | Area (mm^2^)  Frequency (cm^-1^)  FWHM (cm^-1^) |  |  |  |  |
| S-A-3.5 | Area (mm^2^)  Frequency (cm^-1^)  FWHM (cm^-1^) | 59.2 ± 25.1  1088.4 ± 0.1  11.7 ± 0.2 | 56.2 ± 17.6  1088.6 ± 0.2  12.1 ± 0.4 | 43.5 ± 11.4  1086.4 ± 3.2  8.3 ± 5.2 | 1.18 / 0.554 / 2  2.00 / 0.368 / 2  2.00 / 0.368 / 2 |
| S-A-6.5 | Area (mm^2^)  Frequency (cm^-1^)  FWHM (cm^-1^) | 158.8 ± 38.7  1085.8 ± 3.0  8.3 ± 5.4 | 117.5 ± 58.0  1086.6 ± 2.6  7.7 ± 5.5 | 142.8 ± 65.9  1084.3  4.0 | 1.18 / 0.554 / 2  1.40 / 0.497 / 2  1.40 / 0.497 / 2 |
| S-A-14 | Area (mm^2^)  Frequency (cm^-1^)  FWHM (cm^-1^) | 143.5 ± 73.1  1086.4 ± 2.8  7.7 ± 4.8 | 94.1 ± 40.3  1088.1 ± 3.1  7.2 ± 4.7 | 105.8 ± 29.1  1086.1 ± 2.5  7.5 ± 5.2 | 2.09 / 0.352 / 2  0.86 / 0.651 / 2  0.29 / 0.867 / 2 |
| S-L-3.5 | Area (mm^2^)  Frequency (cm^-1^)  FWHM (cm^-1^) | 24.7 ± 6.7  1087.8 ± 0.1  12.0 ± 0.3 | 21.4 ± 8.1  1087.8 ± 0.7  12.4 ± 0.1 | 25.4 ± 7.2  1087.8 ± 0.1  11.9 ± 0.4 | 0.64 / 0.727 / 2  0.00 / 1.000 / 2  3.71 / 0.156 / 2 |
| S-L-6.5 | Area (mm^2^)  Frequency (cm^-1^)  FWHM (cm^-1^) | 201.7 ± 58.7  1086.5 ± 1.2  7.9 ± 5.7 | 179.9 ± 38.4  1086.5 ± 2.8  7.6 ± 5.2 | 158.8 ± 37.5  1088.2 ± 0.2  10.9 ± 0.1 | 1.79 / 0.408 / 2  0.29 / 0.867 / 2  0.29 / 0.867 / 2 |
| S-L-14 | Area (mm^2^)  Frequency (cm^-1^)  FWHM (cm^-1^) | 236.6 ± 6.7  1085.1 ± 1.2  5.9 ± 2.8 | 138.2 ± 13.1  1085.9 ± 3.5  7.7 ± 4.7 | 169.2 ± 51.9  1086.2 ± 2.7  7.3 ± 4.7 | 5.98 / 0.500 / 2  0.29 / 0.867 / 2  1.14 / 0.565 / 2 |
| S-EL-3.5 | Area (mm^2^)  Frequency (cm^-1^)  FWHM (cm^-1^) | 0.9 ± 0.4 | 0.5 ± 0.1 | 0.5 | 3.52 / 0.172 / 2 |
| S-EL-6.5 | Area (mm^2^)  Frequency (cm^-1^)  FWHM (cm^-1^) | 0.5 ± 0.2 | 0.4 ± 0.2 | 0.3 ± 0.1 | 1.65 / 0.437 / 2 |
| N-EL-14 | Area (mm^2^)  Frequency (cm^-1^)  FWHM (cm^-1^) | 0.3 ± 0.2 | 0.2 ± 0.1 | 0.1 | 3.60 / 0.165 / 2 |

**Reference:** 1. Downs R.T. 2006 The RRUFF Project: an integrated study of the chemistry, crystallography, Raman and infrared spectroscopy of minerals. In *Program and Abstracts of the 19th General Meeting of the International Mineralogical Association* (Kobe, Japan).
